# National Hospital-Based Sentinel Surveillance for Cholera in Bangladesh: Epidemiological Results from 2014 to 2021

**DOI:** 10.4269/ajtmh.23-0074

**Published:** 2023-08-14

**Authors:** Md Taufiqul Islam, Sonia Tara Hegde, Ashraful Islam Khan, Md Taufiqur Rahman Bhuiyan, Zahid Hasan Khan, Faisal Ahmmed, Yasmina Ara Begum, Mokibul Hassan Afrad, Mohammad Ashraful Amin, Nabid Anjum Tanvir, Ishtiakul Islam Khan, Zakir Hossain Habib, Ahmed Nawsher Alam, Nigel A. McMillan, Tahmina Shirin, Andrew S. Azman, Firdausi Qadri

**Affiliations:** ^1^ Infectious Diseases Division, International Centre for Diarrhoeal Disease Research, Bangladesh (icddr,b), Dhaka, Bangladesh;; ^2^ School of Medical Science, Griffith University, Gold Coast, Australia;; ^3^ Department of Epidemiology, Johns Hopkins Bloomberg School of Public Health, Baltimore, Maryland;; ^4^ Institute of Epidemiology, Disease Control and Research (IEDCR), Dhaka, Bangladesh

## Abstract

Despite focusing on cholera burden, epidemiologic studies in Bangladesh tend to be limited in geographic scope. National-level cholera surveillance data can help inform cholera control strategies and assess the effectiveness of preventive measures. Hospital-based sentinel surveillance among patients with suspected diarrhea in different sites across Bangladesh has been conducted since 2014. We selected an age-stratified sample of 20 suspected cholera cases each week from each sentinel site, tested stool for the presence of *Vibrio cholerae* O1/O139 by culture, and characterized antibiotic susceptibility in a subset of culture-positive isolates. We estimated the odds of being culture positive among suspected cholera cases according to different potential risk factors. From May 4, 2014 through November 30, 2021, we enrolled 51,414 suspected cases from our sentinel surveillance sites. We confirmed *V. cholerae* O1 in 5.2% of suspected cases through microbiological culture. The highest proportion of confirmed cholera cases was from Chittagong (9.7%) and the lowest was from Rangpur Division (0.9%). Age, number of purges, duration of diarrhea, occupation, and season were the most relevant factors in distinguishing cholera-positive suspected cases from cholera-negative suspected cases. Nationwide surveillance data show that cholera is circulating in Bangladesh and the southern region is more affected than the northern region. Antimicrobial resistance patterns indicate that multidrug resistance (resistance to three or more classes of antibiotics) of *V. cholerae* O1 could be a major threat in the future. Alignment of these results with Bangladesh’s cholera-control program will be the foundation for future research into the efficacy of cholera-control initiatives.

## INTRODUCTION

Cholera, an epidemic-prone diarrheal disease, is considered a major public health problem in the world, particularly in low- and middle-income countries.^1^^,^^2^ Globally, more than one billion people are at risk of cholera in endemic settings and an estimated 1.3–4.0 million cases and 21,000–143,000 deaths due to cholera occur annually.^3^ Cholera causes epidemics throughout the world in populations with limited access to water and sanitation infrastructure and occurs regularly in much of South Asia and sub-Saharan Africa.^4^^,^^5^

Previous cholera pandemics, as well as much of the genetic diversity in the current seventh pandemic, are thought to have originated in areas around the Bay of Bengal.^4^^,^^6^ To date, no precise estimates of the cholera burden in Bangladesh have been made because of limited systematic clinical surveillance. However, one estimate suggests that 66 million people are at risk of cholera, with a mean annual incidence of 1.64 per 1,000 persons and 4,000 annual deaths.^7^ Another estimate based on a national serologic survey conducted in 2015 suggests that each year about one in six people nationally are exposed to the primary causative bacteria of cholera, *Vibrio cholerae* O1.^8^

The Global Task Force on Cholera Control (GTFCC) launched a global strategy in 2017, “Ending Cholera—A Global Roadmap to 2030,” to reduce cholera mortality by 90% by 2030. With the commitment of cholera-prone countries, technical partners, and donors, as many as 20 countries including Bangladesh plan to eliminate cholera as a major public health threat by 2030.^9^ A long-term multi-sectoral prevention and control strategy ensuring adequate access to the cholera vaccine, water, and sanitation and social mobilization for health and hygiene promotion, surveillance, and rapid and appropriate case management are essential for reducing the morbidity and mortality of cholera in both endemic and epidemic contexts.

Standardized cholera surveillance is important for estimating incidence and allocating scarce cholera control resources, tracking changes in *V. cholerae* phenotypes (e.g., antimicrobial resistance), and monitoring the impact of the interventions planned for the cholera endgame.^10^ Using existing healthcare facilities, the International Centre for Diarrhoeal Disease Research, Bangladesh (icddr,b), in collaboration with the Institute of Epidemiology Disease Control and Research (IEDCR), established a nationwide, sentinel surveillance system in Bangladesh in 2014 to evaluate the epidemiological, seasonal, and geographic patterns of the disease.^11^ The purpose of this manuscript is to describe an epidemiological summary of cholera, including the effects of predisposing factors such as patient age, geographic distribution of the disease, and antimicrobial resistance (AMR) patterns during the May 2014–November 2021 period.

## MATERIALS AND METHODS

### Case definitions.

We defined a suspected cholera case as an individual of any age presenting with acute watery diarrhea, although diarrhea is defined differently for those less than 2 months old and those 2 months and older. For those less than 2 months old, acute watery diarrhea was defined as stool habits that differed from the norm in terms of intensity (more purging than normal) or type of stool (more water than fecal matter). For those older than 2 months, acute watery diarrhea was defined as having three or more loose or watery stools in the preceding 24 hours or three or fewer loose/liquid stools causing dehydration.^11^ Our suspected case definition excluded those with bloody diarrhea. A confirmed cholera case was a suspected case with *V. cholerae* O1 or O139 confirmed by microbiological culture. Diarrhea can be fatal if it is not treated immediately and adequate fluid replacement is not administered. Early recovery may be possible when immediate rehydration can be achieved.

### Surveillance system.

The nationwide sentinel surveillance network included both government health facilities and private medical college health facilities that provide care at a minimum cost. Initially, the surveillance included 10 sentinel sites in 2014; this was extended to 22 sites in 2016. In all, 13 districts, six subdistricts, two tertiary-level hospitals, and the Bangladesh Institute of Tropical and Infectious Diseases were included, as previously described.^11^ Because of funding constraints, the number of facilities was reduced to 16 sites from 2020 onward. The surveillance team at each site was composed of one study physician, one nurse, and one medical technologist, all of whom were existing local hospital staff. In addition, one trained field attendant from the icddr,b worked at each sentinel site.

Given our funding, we aimed to enroll 20 suspected cholera cases each week from the diarrheal inpatient ward, outpatient care, and emergency ward of each sentinel site. We enrolled suspected cholera cases 5 days a week, with a goal of enrolling the first two who were younger than 5 years and the first two older than 5 years each day. When we were unable to enroll 20 suspected cases in a given week, we extended surveillance 1 additional day to try to meet this target (Supplemental Figure 1). In addition, when there were too few suspected cases in one age group, we overenrolled in the other age group.

From suspected cholera participants, we collected informed written consent, sociodemographic and clinical data, and a biological sample (stool or rectal swab when the patient was no longer passing stool during enrollment). Questions on the participant survey included sociodemographic factors (age, gender, education, occupation), clinical factors (duration of diarrhea, number of diarrheal episodes [or purging] in the last 24 hours, nature of the stool, vomiting, dehydration status, abdominal cramps, fever), potential exposure histories, and history of antibiotic use for the current diarrheal episode. The samples were then transferred to the icddr,b and IEDCR laboratories in Dhaka using Cary-Blair (Thermo Fisher Scientific, Waltham, MA) transport medium twice monthly. We used Cary-Blair transport medium because it maintains the viability of *V. cholerae* in the sample for more than 1 month.^12^^,^^13^

### Laboratory testing.

Samples were plated over taurocholate-tellurite gelatin agar and incubated overnight at 37 °C to identify *V. cholerae*. For enrichment, samples were inoculated in alkaline peptone water and incubated for a further 18–24 hours.^14^ Monoclonal antibodies unique for *V. cholerae* O1 (Ogawa and Inaba) as well as O139 serogroups were used to categorize suspected colonies.^15^^,^^16^

A randomly selected subsample (about 20% of the positive cases) underwent antibiotic susceptibility testing. For the entire study period, we tested the following antibiotic classes: tetracyclines (doxycycline), macrolides (erythromycin, azithromycin), and fluoroquinolones (ciprofloxacin). At the end of 2017, we added beta-lactams including penicillin (ampicillin) and cephalosporins (ceftriaxone, cefixime). Bacterial susceptibility to antimicrobial agents was determined with the disc diffusion method by measuring zone size (in millimeters) following the guidelines of the Clinical and Laboratory Standards Institute,^17^ which included the inhibition distances for all antimicrobials, using commercially available antibiotic discs (Oxoid, Basingstoke, United Kingdom). *Escherichia coli* American Type Culture Collection 25922 susceptible to all antimicrobials was used as a control strain for susceptibility studies.

### Statistical analysis.

We described the frequency and percentage of different sociodemographic factors and clinical symptoms among all patients with suspected cholera and the proportion of cholera cases by month, division (the largest administrative unit), and age group. We examined the surveillance data by specific age strata (< 5, 5–17, 18–45, 46–60, and > 60 years) to evaluate the risk of cholera positivity among different groups; given our enrollment strategy, we investigated risk in the < 5-year-old group versus other age groups. We set the present age strata (< 5 and > 5 years) of the patients because diarrheal diseases are one of the leading causes of illness and death among children < 5 years of age.^18^^,^^19^ We also examined the number of suspected and confirmed cases among those < 2 years old, as the incidence of rotavirus is higher in this age group locally.^20^

To measure the difference between proportions of suspected and confirmed cholera cases, a bivariate analysis of various risk factors is presented as the frequency, percentage, and χ^2^ statistic. Given our sampling scheme, we estimated the odds of being a confirmed cholera case on the basis of different potential risk factors, with a binomial distribution controlling for individual clustering by sentinel site and stratified by age group (< 5 years, ≥ 5–17 years, and ≥ 18 years). We assumed an independent correlation structure, and 95% CIs were calculated using robust standard errors. We conducted univariate analyses to estimate the crude odds ratio of cholera given each factor, which included sociodemographic factors, clinical factors, exposure history, history of antibiotic use prior to enrollment, and season (March–June: pre-monsoon; July–August: monsoon; September–October: post-monsoon; November–February: other). Given the large number of statistical tests, we corrected *P* values for multiple comparisons by using the Bonferroni correction method; we considered associations as significant only after the correction.^21^

We used a random forest classification model to understand the predictive value of season, sociodemographic factors, clinical features, and exposure measures to predict whether a suspected cholera case would be culture confirmed. We excluded antibiotic use history because antibiotic use in the 24 hours prior to presentation could lead to false-negative results by culture.^22^ We used 5-fold cross-validation to evaluate the model performance and then ran the model on the full data to understand the predictive importance of all variables used. Model fit was determined by area under the cross-validated receiver operator characteristic curve (cvAUC).

We conducted a descriptive analysis of the serotypes identified over the course of the surveillance period and the AMR patterns over time by geography and serotype. We examined resistance to different classes of antibiotics and multidrug resistance (MDR; resistance to three or more classes) and described the distinct AMR phenotypes by serotype. All analyses were carried out with R version 4.1.0 using the *gee* package to perform the risk factor analysis and the *randomForest* package version 4.7–1.1 for the random forest analysis.

## RESULTS

### Suspected cholera.

From May 4, 2014 through November 30, 2021, we enrolled 51,414 suspected cases from our sentinel surveillance sites. There were slightly more male participants enrolled (54%) than female participants (Table 1). Among the participants with suspected cholera, 41.8% were < 2 years of age, 6.1% were 2–4 years old, 2.3% were 5–9 years old, and 49.8% were ≥ 10 years old (Supplemental Figures 2 and 3). Of the suspected cases, 28% were < 5 years old (per sampling design), and 97% of suspected cases were from inpatient departments. Twenty-two percent of suspected cholera cases were housewives and 6.3% were service holders (who used in the organization). For the suspected cholera cases, the median number of purges in the last 24 hours before presentation was 15 (interquartile range [IQR]: 10–20), and the median history of diarrhea was 2 days (IQR: 2–3). Overall, 44% of suspected cases reported taking antibiotics for their symptoms prior to attending the hospital, with the highest proportion (72%) recorded in the Mymensingh Division and the lowest in Barisal (23%) (Table 1, Supplemental Table 1).

**Table 1:** Distribution of demographics for suspected cholera and confirmed cholera for the 2014–2021 period across all sentinel sites in Bangladesh

Characteristic	Suspected cholera cases, *N* = 51,414*	Culture negative, *N* = 48,743*	Culture positive, *N* = 2,671*	*P* value†
Season				
Pre-monsoon	14,918 (29%)	13,858 (28%)	1,060 (40%)	< 0.001
Monsoon	7,164 (14%)	6,755 (14%)	409 (15%)	
Post-monsoon	9,627 (19%)	9,032 (19%)	595 (22%)	
Other	19,705 (38%)	19,098 (39%)	607 (23%)	
Age (years)				
< 5	24,609 (48%)	24,230 (50%)	379 (14%)	< 0.001
5–17	2,510 (4.9%)	2,228 (4.6%)	282 (11%)	
18–45	16,816 (33%)	15,262 (31%)	1,554 (58%)	
46–60	3,958 (7.7%)	3,715 (7.6%)	243 (9.1%)	
61+	3,521 (6.8%)	3,308 (6.8%)	213 (8.0%)	
Gender				
Female	23,840 (46%)	22,579 (46%)	1,261 (47%)	0.4
Male	27,574 (54%)	26,164 (54%)	1,410 (53%)	
Occupation				
Service holder	3,534 (6.9%)	3,079 (6.3%)	455 (17%)	< 0.001
Housewife	11,590 (23%)	10,808 (22%)	782 (29%)	
Agriculture worker	2,220 (4.3%)	2,081 (4.3%)	139 (5.2%)	
Businessman	2,186 (4.3%)	1,996 (4.1%)	190 (7.1%)	
Labor/worker/driver	1,880 (3.7%)	1,636 (3.4%)	244 (9.1%)	
Student and unemployed	3,836 (7.5%)	3,493 (7.2%)	343 (13%)	
Child (up to 10 years old)	25,785 (50%)	25,292 (52%)	493 (18%)	
Other	383 (0.7%)	358 (0.7%)	25 (0.9%)	
Education status				
Illiterate	7,034 (14%)	6,523 (13%)	511 (19%)	< 0.001
Up to class VIII	12,565 (24%)	11,368 (23%)	1,197 (45%)	
Secondary and above	6,268 (12%)	5,750 (12%)	518 (19%)	
Other (child, etc.)	25,547 (50%)	25,102 (51%)	445 (17%)	
Duration of diarrhea				
Median (IQR)	2 (1–3)	2 (1–3)	2 (1–2)	< 0.001
Number of purgings in last 24 hours				
Median (IQR)	15 (10–20)	15 (10–20)	16 (12–22)	< 0.001
Nature of stool				
Loose watery	36,073 (70%)	34,327 (70%)	1,746 (65%)	< 0.001
Rice watery	14,267 (28%)	13,366 (27%)	901 (34%)	
Formed	1,074 (2.1%)	1,050 (2.2%)	24 (0.9%)	
Vomiting	33,048 (64%)	31,036 (64%)	2,012 (75%)	< 0.001
Dehydration				
No	9,457 (18%)	9,025 (19%)	432 (16%)	< 0.001
Some	34,151 (66%)	32,686 (67%)	1,465 (55%)	
Severe	7,806 (15%)	7,032 (14%)	774 (29%)	
Abdominal cramp	29,924 (58%)	28,063 (58%)	1,861 (70%)	< 0.001
Fever	29,497 (57%)	28,237 (58%)	1,260 (47%)	< 0.001
Tap water	12,634 (25%)	11,607 (24%)	1,027 (38%)	< 0.001
Tube well	42,457 (83%)	40,525 (83%)	1,932 (72%)	< 0.001
Bottled water	4,434 (8.6%)	4,169 (8.6%)	265 (9.9%)	0.014
Water treated by boiled/filtered/chemical	7,818 (15%)	7,300 (15%)	518 (19%)	< 0.001
Take food from roadside	14,166 (28%)	13,323 (27%)	843 (32%)	< 0.001
Take food from large gatherings	5,413 (11%)	5,043 (10%)	370 (14%)	< 0.001
Any one of neighbors has the same disease	7,741 (15%)	7,259 (15%)	482 (18%)	< 0.001
Use of antibiotic for current illness	22,875 (44%)	21,822 (45%)	1,053 (39%)	< 0.001

* *n* (%).

† Pearson’s χ^2^ test.

### Confirmed cholera.

**Figure 1.**
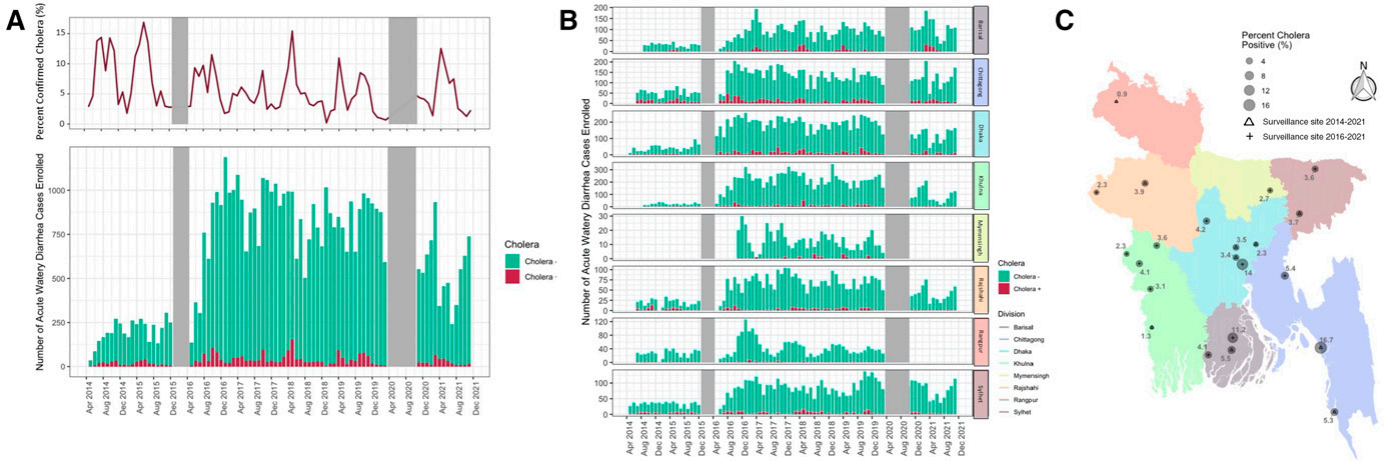
(**A**) A bar plot of the number of enrolled suspected cholera cases disaggregated by *Vibrio cholerae* culture positivity across the sentinel sites over the period of 2014 to 2021 and a line plot of the percentage of culture-confirmed cholera cases over the same period. The first pause in surveillance was due to a pause in funding (grey area from December 2015 to April 2016), and the second pause in surveillance was due to the COVID-19 pandemic (from March 2020 to October 2020). (**B**) A bar plot of the number of enrolled suspected cholera cases disaggregated by *V. cholerae* culture positivity across sentinel sites grouped by division over the period of 2014 to 2021. (**C**) Map of divisions of Bangladesh and the cholera positivity (proportion of suspected cholera cases that were confirmed by culture) by sentinel sites calculated for the entire study period. Each sentinel site is indicated by a symbol to signify the years of data captured, and the size of each circle denotes the cholera positivity. The color of each division on the map matches the color of each division in Figure 1B.

We confirmed *V. cholerae* O1 in 5.2% of suspected cases (2,671/51,414), and one sample was positive for *V. cholerae* O139 through microbiological culture, with significant differences in positivity between age groups (Table 1). In aggregate, cholera positivity tended to have two annual peaks, the first during the pre-monsoon season (March–June) and the second during the post–monsoon season (September–October), with a higher average cholera positivity occurring during the spring peak in the southeast part of the country and substantial variability between individual locations (Supplemental Figure 4). Among confirmed cholera cases, 58% were 18–45 years of age, 29% were housewives, and 17% were service holders (Table 1). The median number of purges in the last 24 hours before presentation was 16 (IQR: 12–22), and the median history of diarrhea was 2 days (IQR: 1–2) for the confirmed cholera cases (Supplemental Table 2). The highest proportions of confirmed cholera cases were from Chittagong (9.7%), Dhaka (7.4%), and Barisal (5.6%), and the lowest proportions were recorded in Khulna (2.8%) and Rangpur Division (0.9%) (Figure 1, Supplemental Table 3, and Supplemental Figure 5). The southern sentinel sites had higher cholera positivity on average across all months and seasons than the northern sentinel sites (Supplemental Figure 4A and B).

We observed three distinct periods when a single serotype of *V. cholerae* O1 was dominant across the country: From May 2014 to May 2016, nearly all samples were Ogawa (90%); from May 2016 to May 2018, nearly all were Inaba (96%); and from May 2018 to March 2020, nearly all were Ogawa (90%). After the pandemic period, there was co-circulation of the serotypes with 39% Ogawa and 61% Inaba (Supplemental Figure 6).

### Predictors of cholera positivity.

**Figure 2.**
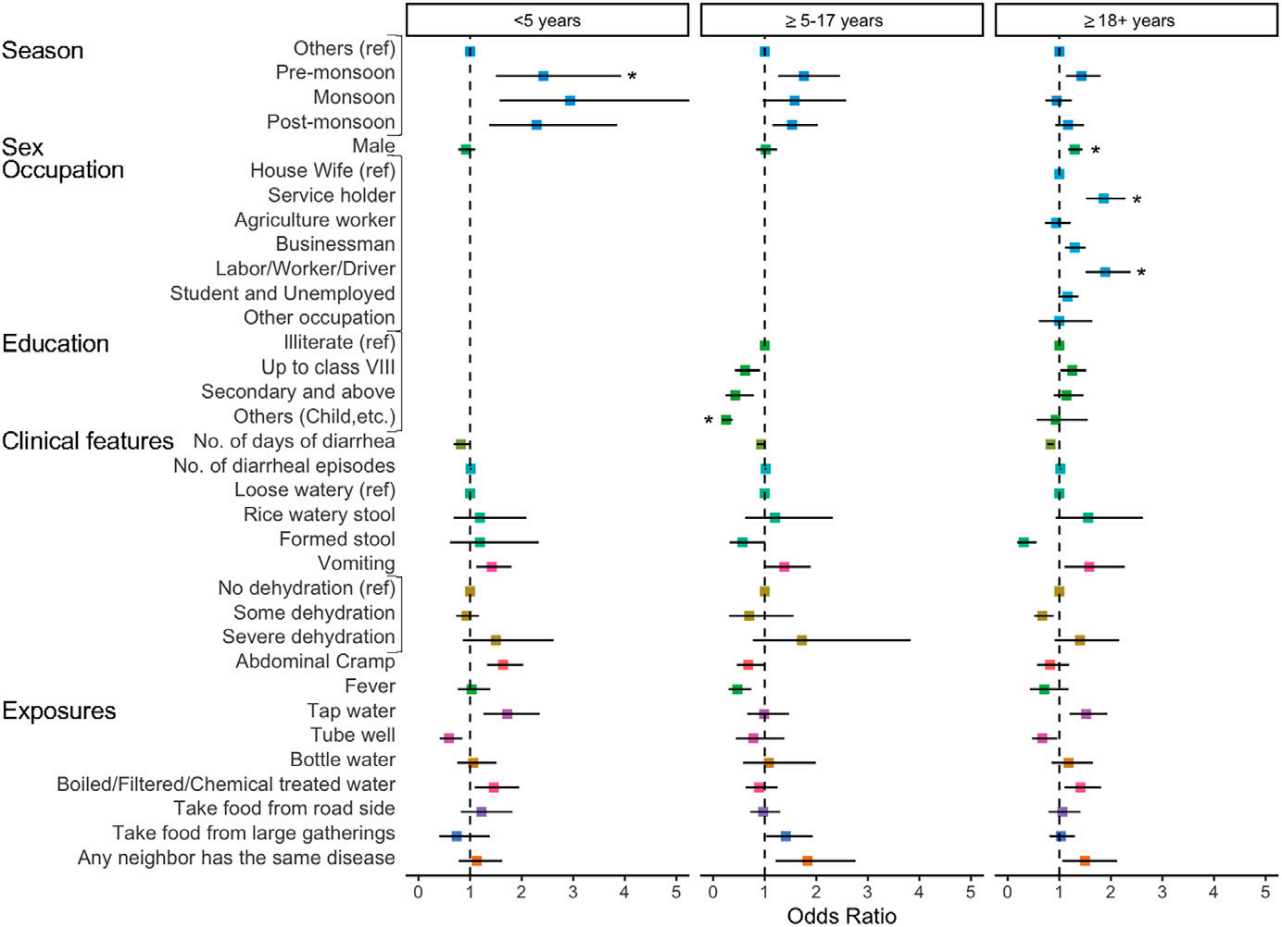
Forest plot of the crude odds ratios of being a confirmed cholera case given the different seasons, sociodemographic variables, clinical features, and possible exposures across the sentinel sites for the entire study period and stratified by age using the following groups: < 5 years of age, ≥ 5–17 years of age, and ≥ 18 years of age. Age groups were chosen to match the enrollment scheme. The 95% CIs were calculated using robust standard errors from the GEE models. The asterisk (*) indicates significant associations after the Bonferroni correction was applied to *P* values. GEE = generalized estimating equation; ref = reference.

We explored clinical, demographic, and ecological factors that helped to distinguish culture-positive suspected cases from culture-negative suspected cases. In age-stratified univariate analyses, we found that the odds of a suspected case having *V. cholerae* O1/O139 isolated in stool during pre-monsoon season (March–June) was 2.4 (95% CI: 1.50–3.93) times higher than the other season (November–February) in children under 5 years old. Although the pre-monsoon season was associated with greater odds of being a confirmed cholera case for both those aged 5–17 years and those > 18 years of age, this was not statistically significant. For children < 5 years old, the odds of being confirmed with cholera during the monsoon season (July–August) was 2.9 (95% CI: 1.57–5.51) times higher and during the post-monsoon season (September–October) was 2.3 (95% CI: 1.37–3.85) times higher than the other season, though these were not statistically significant after accounting for multiple tests. The odds of being a confirmed cholera case was 1.4 (95% CI: 1.12–1.80) times higher among children < 5 years old who presented with vomiting (Figure 2, Supplemental Table 4).

Among adults, the odds of testing positive for cholera was significantly higher among males than among females (1.3; 95% CI: 1.17–1.45). Service holders (1.8; 95% CI: 1.52–2.28) and day laborers, workers, or drivers (1.8; 95% CI: 1.51–2.38) were also significantly more likely than housewives to be a confirmed cholera case. Although not significant after the Bonferroni correction, having tap water as the primary source of water in the household was associated with elevated odds of being a confirmed cholera case for children < 5 years old and adults.

**Figure 3.**
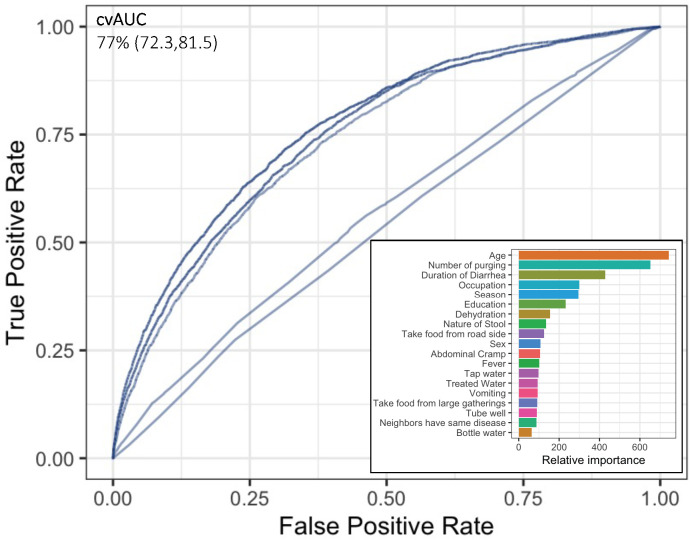
The cvAUCs and variable of importance from random forest models. cvAUCs = cross-validated receiver operating characteristic curves.

When combining all 19 measured attributes for each suspected case in a random forest classification model to predict who would be a confirmed cases, we found a cvAUC of 77% (95% CI: 72.3–81.5%). The most influential attributes in this model were age, number of purges, duration of diarrhea, occupation, and season (Figure 3). At the point that jointly maximized model sensitivity and specificity (Youden index), the model correctly classified 70.8% of true culture-confirmed cholera cases (sensitivity) and falsely classified 31% of suspected cases as culture confirmed (1 − specificity).

### Bacterial analyses.

**Figure 4.**
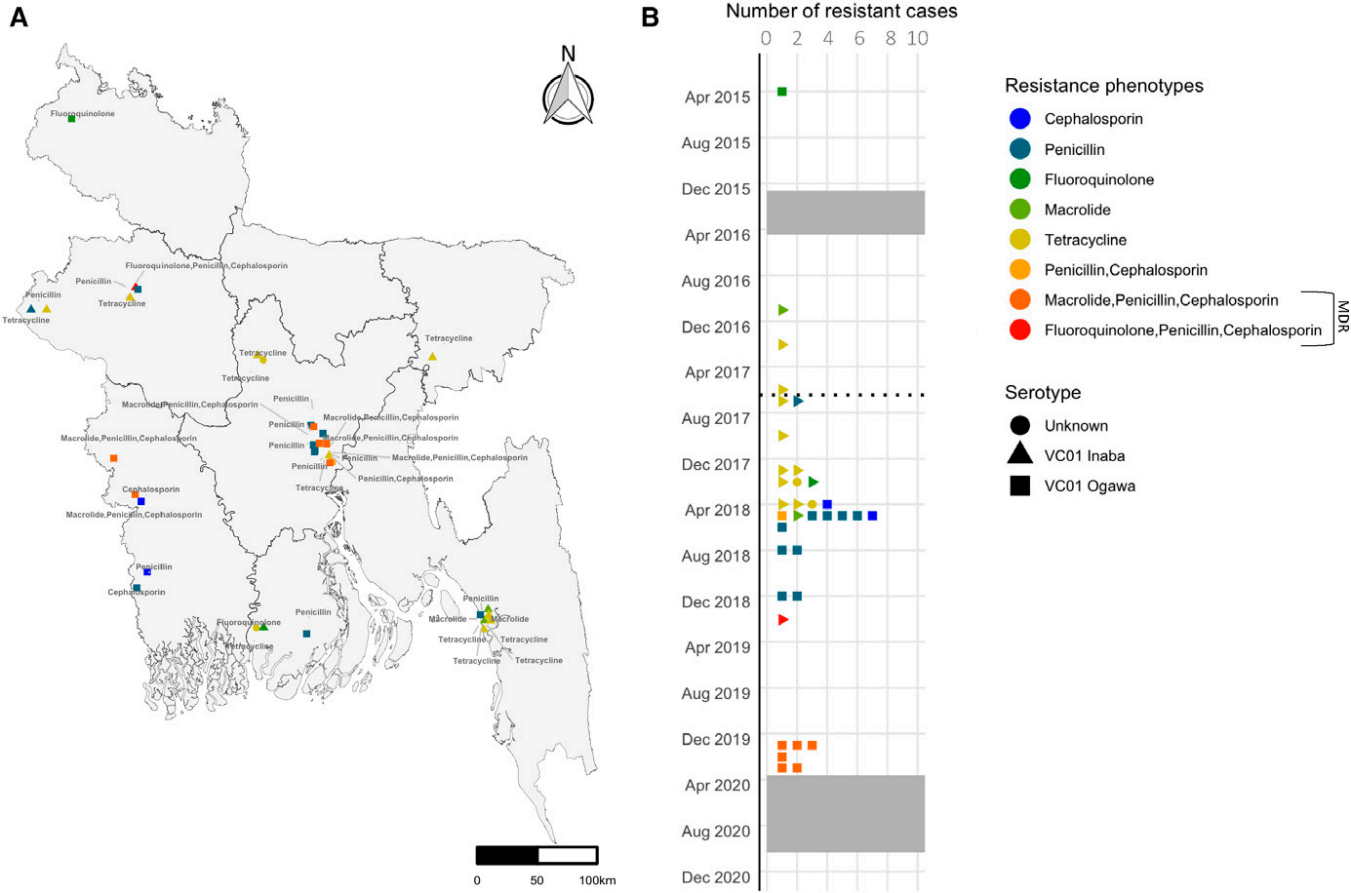
Unique AMR phenotypes by (**A**) antibiotic class and serotype over time and geographic location (2014–2021); (**B**) the unique MDR phenotypes indicate simultaneous resistance to ceftriaxone, cefixime, ampicillin, ciprofloxacin and ceftriaxone, cefixime, ampicillin, erythromycin, and azithromycin. AMR = antimicrobial resistance; MDR = multidrug resistance.

We tested 581 isolates for AMR, and 35 (6%) were resistant to at least one of the antibiotics tested. There were nine unique AMR phenotypes (Figure 4). Among the 35 resistant isolates, one was resistant to two antimicrobial classes and seven (20%) were multidrug resistant (resistance to three or more classes); 1.2% of all isolates (7/581) were MDR. Of the seven MDR cholera cases, six were *V. cholerae* O1 Ogawa cases that were resistant to all the macrolides, penicillin, and cephalosporins tested (erythromycin, azithromycin, ampicillin, ceftriaxone, cefixime) (Figure 4). These cases occurred after the start of the COVID-19 pandemic in the Dhaka and Khulna divisions. The one other MDR case was of the *V. cholerae* O1 Inaba serotype and occurred in 2019 in Rajshahi resistant to all the fluoroquinolones, penicillin, and cephalosporins tested (ciprofloxacin, ampicillin, ceftriaxone, cefixime).

Different AMR patterns were detected over the surveillance period. In 2018, intermediate resistance was 24.3% to doxycycline and 9.5% to ciprofloxacin, which was reduced to 0% in the following years (Supplemental Figure 7). The cephalosporins, ceftriaxone and cefixime, and azithromycin were largely sensitive over the years.

## DISCUSSION

Cholera is a significant public health issue in Bangladesh, where it is endemic. The results generated from this surveillance study illustrate that cholera has persisted in Bangladesh from 2014 to the present; in addition, distinct differences between suspected and confirmed cholera cases include age, number of purges, duration of diarrhea, occupation, and season. Although cholera is widespread across the nation, there were significant differences within and across geographic locations, including factors such as AMR, which necessitates continued national surveillance and systematic testing of cholera. Importantly, Chittagong and Dhaka remain critical areas for cholera risk, having the highest observed cholera positivity among the sentinel sites. Cholera is still a significant cause of morbidity and mortality in areas where public health facilities are lacking. Because *V. cholerae* can grow well in salty and warm waters, the southern part of the country, especially the coastal areas, are vulnerable and most often affected. Bangladesh is also a flood-prone country, which directly alters the quality of water and triggers cholera transmission. In addition, cases and mortality rates were higher than usual in 2021 in several regions. High morbidity and even fatalities were documented in the Barisal Division, Kishorganj, Noakhali, Gopalganj, Bandarban, and Chattogram during these outbreaks.^23^

We observed that among suspected cholera cases, older children were more likely to be a confirmed cholera case. The WHO emphasizes that in endemic settings individuals > 2 years old who have acute watery diarrhea and severe dehydration should be suspected of having cholera.^24^ As we showed here, the majority of suspected cases in those younger than 5 years were in the < 2 years of age category, and these children had the highest number of non-cholera diarrheal episodes, the bulk of which were likely caused by rotavirus.^11^^,^^25^^–^^28^

Our generalized estimating equation model showed that the odds of having cholera are high among those < 18 years of age and particularly among adults who are service holders, day laborers, and drivers. Other cholera risk factors include young age, having a low level of education, or being a household member who was in contact with a cholera case during the monitoring period. These findings are similar to those of previous studies conducted in Bangladesh.^29^^,^^30^

With 7 years of data, our study indicates that cholera cases occurred throughout the year in Bangladesh, although with different levels of frequency. This included those younger than 5 years old (in all three seasons: pre-, during, and after the monsoon) and those 5–17 years old (except during the monsoon). Children younger than 5 years were more likely to have *V. cholerae* O1 in the stool during the pre-monsoon season (March–June) than in other seasons (November–February). A previously published paper from Bangladesh revealed that cholera had two distinct peaks in different geographical locations.^11^^,^^31^ The correlation between hydroclimatological changes and cholera in Bangladesh should be evaluated in future studies.^32^

As indicated previously, the duration of diarrhea, number of diarrheal episodes, stool type, and dehydration were important variables for determining whether a patient with acute watery diarrhea had culture-confirmed cholera.^33^ However, along with other sociodemographic variables such as age and occupation, exposures, and season, such symptoms cannot sufficiently predict a confirmed cholera case alone, as shown by our random forest classification model. Because laboratory testing is either unavailable or prohibitively expensive in low-resource settings, physicians may find a sensitive clinical predictive model useful (e.g., for appropriate antibiotic use). More work could be done on refining clinical case definitions for different settings or populations. There are few analyses on the prediction of the etiology of pediatric diarrhea in low-resource settings to inform clinical decision-making which can be replicated for all age groups.^34^

Distinct antimicrobial-resistant patterns were observed for ampicillin, ciprofloxacin, ceftriaxone, erythromycin, azithromycin, and doxycycline, and the fluctuation of antimicrobial-resistant patterns that were detected over time but were consistent across space in this study was similar to other findings from Bangladesh.^35^ Over the years, several antimicrobials, including tetracyclines, fluoroquinolones, and azithromycin, have been effectively used in Bangladesh for the treatment of cholera in patients seen at healthcare facilities.^36^ Therefore, such consistent patterns across Bangladesh may be due to the same kind of antibiotic being used across the country; however, the spatially heterogenous emergence of MDR *V. cholerae* O1 suggests that the inappropriate use of antibiotics likely occurs prior to seeking care as well, which was also found in another study.^35^ All resistant isolates tested after the COVID-19 pandemic had begun were MDR, an indication that MDR *V. cholerae* O1 may become more prevalent as a result of self-guided treatment regimens in the absence of accessible healthcare. Of note, resistance to cefixime was observed only among MDR cases that were also resistant to ceftriaxone, the other cephalosporin tested, and ampicillin. Further, there appear to be differential resistance patterns by serotype, which suggests that active monitoring of *V. cholerae* O1 may be critical for determining which first-line antibiotics should be administered to cholera patients in the hospital to reduce both morbidity and mortality. These data support the premise that antimicrobial-resistant *V cholerae* O1 may become a major public health concern.

The surveillance system has several limitations. First, the surveillance system was confined to 22 sentinel sites, and later on, these were reduced to 16 sites because of a funding shortage. Setting up a higher-density surveillance network at varied levels of the healthcare system, including pharmacies and traditional healers, could improve our estimates of cholera incidence to determine both low- and high-incidence areas and to aid future implementation of interventions to reach the 2030 roadmap goal of ending cholera. Pharmacies and other nontraditional drug sellers are commonly visited by individuals with suspected cases of cholera,^37^^,^^38^ and they sell diarrhea-related products, including antibiotics; thus, missing such cases could lead to underestimation of the true disease incidence. More important, we could not estimate cholera incidence in this analysis because we did not have the line-listing of all suspected cholera cases; our surveillance system captured and tested only a fraction of all medically attended suspected cholera cases, and we thus assumed that the selected suspected cases were representative of all patients attending a sentinel site with complaints of acute watery diarrhea. However, we enrolled the maximum number of patients from the inpatient department, as this was convenient for sample collection, which may have introduced a sampling bias. Second, we used culture as the diagnostic of choice for cholera confirmation, though this assay has reduced sensitivity when antibiotic use is high, thereby leading to underestimates of cholera burden. Third, though we tested only a subset of samples for antimicrobial susceptibility, the age and geographic distribution of samples selected for testing accurately reflected the patient population. Fourth, we did not follow up with participants to collect mortality data. Lastly, we did not collect data related to environmental surveillance, such as data on cleanliness, sanitation in local residential areas, or sources of potable water.

Because Bangladesh is a cholera endemic country, the establishment and continuation of surveillance activities give a key visual on any cholera outbreak in different areas of the country. They also help local and national governments track trends in cholera cases and initiate preparedness plans and public health response and interventions. Moreover, the data generated from this surveillance provided evidence of high-risk priority areas and helped the Government of Bangladesh decide to conduct mass cholera vaccination campaigns in the affected areas. In 2020, Bangladesh launched an oral cholera vaccination program in the high-risk areas of Dhaka city as a preventive campaign, and in 2022 another reactive campaign was conducted in affected areas of urban Dhaka. Since the introduction of the oral cholera vaccine program in Bangladesh, approximately 10.5 million vaccine doses have been delivered to different areas of Dhaka city that were highly affected, as well as in Cox Bazar, including the host community and Rohingya people. In addition, the surveillance system is geographically widespread across all eight divisions of Bangladesh, which has allowed us to closely monitor the changing cholera burden over time with the help of well-trained field and laboratory staff and to foster a collaborative partnership between the icddr,b and IEDCR (government partners). The insights obtained from this surveillance are valuable for designing and monitoring the progress of evolving prevention strategies to achieve the target of the GTFCC’s global roadmap to end cholera.

Providing oral cholera vaccine (OCV) and even a safe water supply are the most important strategies to protect communities from cholera over the long term in endemic settings such as Bangladesh and elsewhere. Results from this study and our ongoing surveillance system will aid public health experts and government agencies in analyzing the effects of initiatives such as the OCV campaigns by evaluating how the risk of cholera and the cholera burden change over time. The GTFCC should prioritize adequate surveillance systems in targeted countries with systematic confirmatory testing schemes, particularly as the COVID-19 pandemic persists and healthcare-seeking behaviors have changed, resulting in fewer medically attended cases, higher antibiotic use, and likely increased AMR. Although an increase in handwashing and hygiene in response to the COVID-19 pandemic is expected to decrease food and waterborne infections such as cholera, evidence generated from surveillance systems is necessary to monitor such claims and help achieve long-term cholera control.

We were able to identify priority areas for vaccination and awareness campaigns and were also able to focus our endeavors on areas where the cholera outbreak was most severe. However, more work still needs to be done to achieve the target of ending cholera by 2030 through an integrated, multi-sectorial approach.

## Supplemental Materials


Supplemental materials
