# Supplementary material for: National Hospital-Based Sentinel Surveillance for Cholera in Bangladesh: Epidemiological Results from 2014 to 2021
Source: Am J Trop Med Hyg. 2023 Aug 14;109(3):575–83. doi: 10.4269/ajtmh.23-0074 (PMC10484282; doi:10.4269/ajtmh.23-0074)
Supplement: Supplementary file 1 [file tpmd230074.SD1.pdf]

Supplemental Figure 1. Selection of suspected cases from all sentinel sites for the entire study period from 2014 to 2021

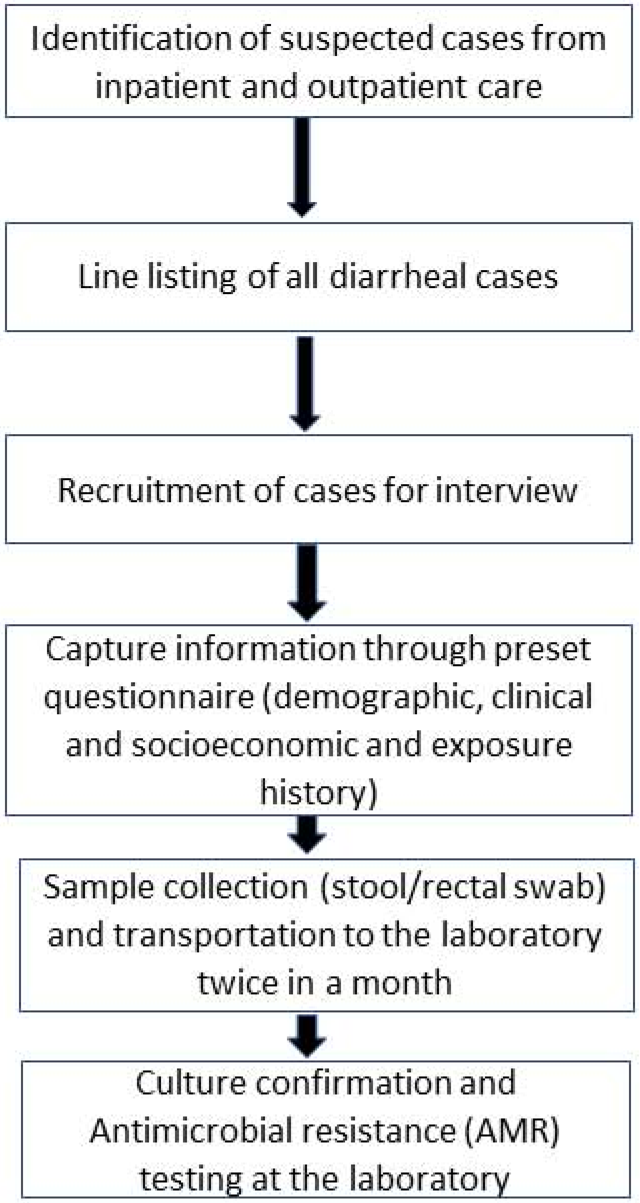

Supplemental Figure 2. Suspected and confirmed cholera cases over the period of 2014-2021 in the population pyramid of Bangladesh

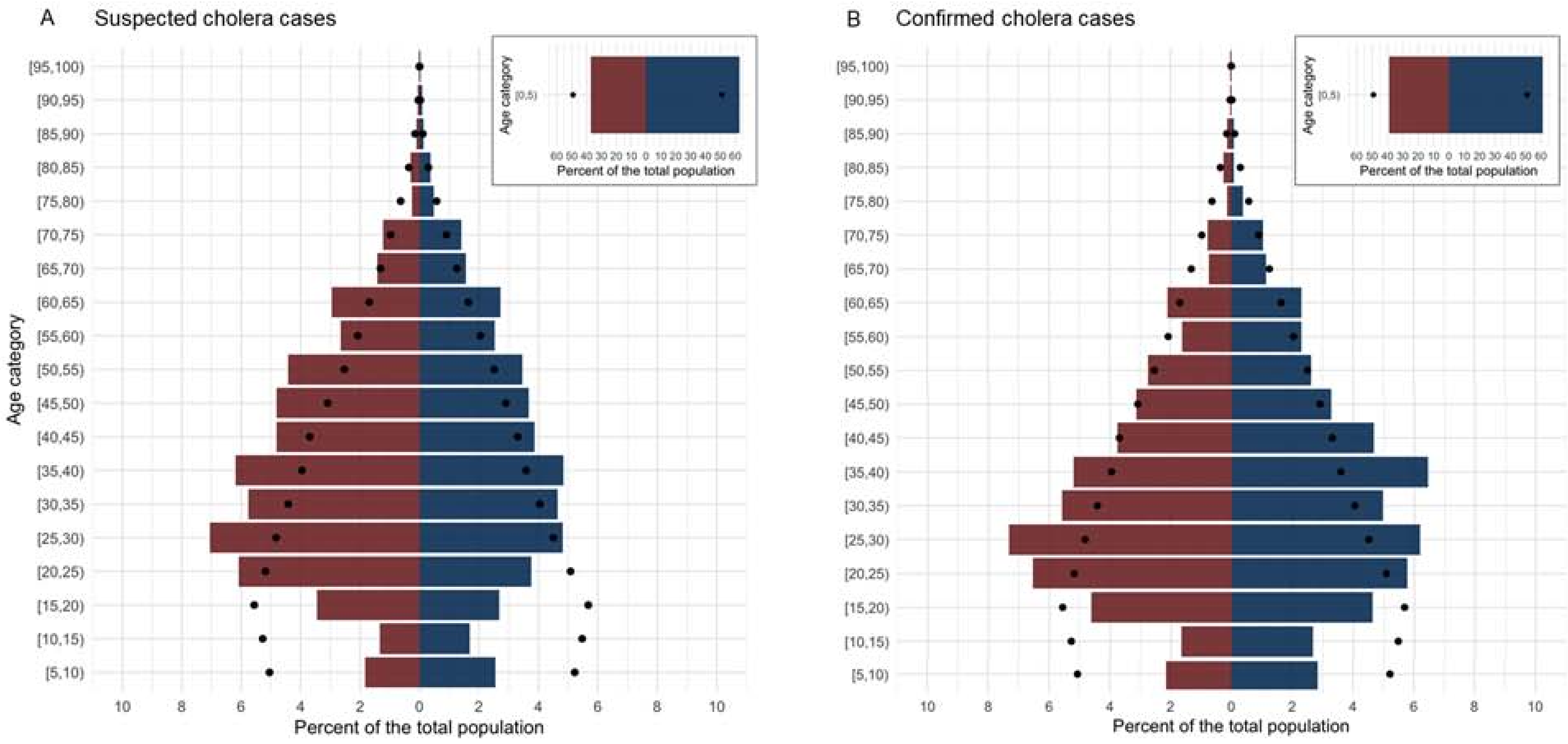

Supplemental Figure 3. (A) Bar plot with the distribution of suspected cholera cases and culture-confirmed cholera cases by age groups: <2, 2-4, 5-17, 18-45, 46-60, and >60 years for the period of 2014 to 2021. The line graph denotes the percent positivity of the culture-confirmed cholera cases for the same age groups. (B) Month-wise distribution of suspected cholera cases by age groups (<2, 2-4, 5-17, 18-45, 46-60, and >60 years) for the entire study period. For both of the figures the first break in surveillance was due to a pause in funding (the grey area from December 2015 to April 2016), and the second break-in surveillance was due to the COVID-19 pandemic (from March 2020 to October 2020)

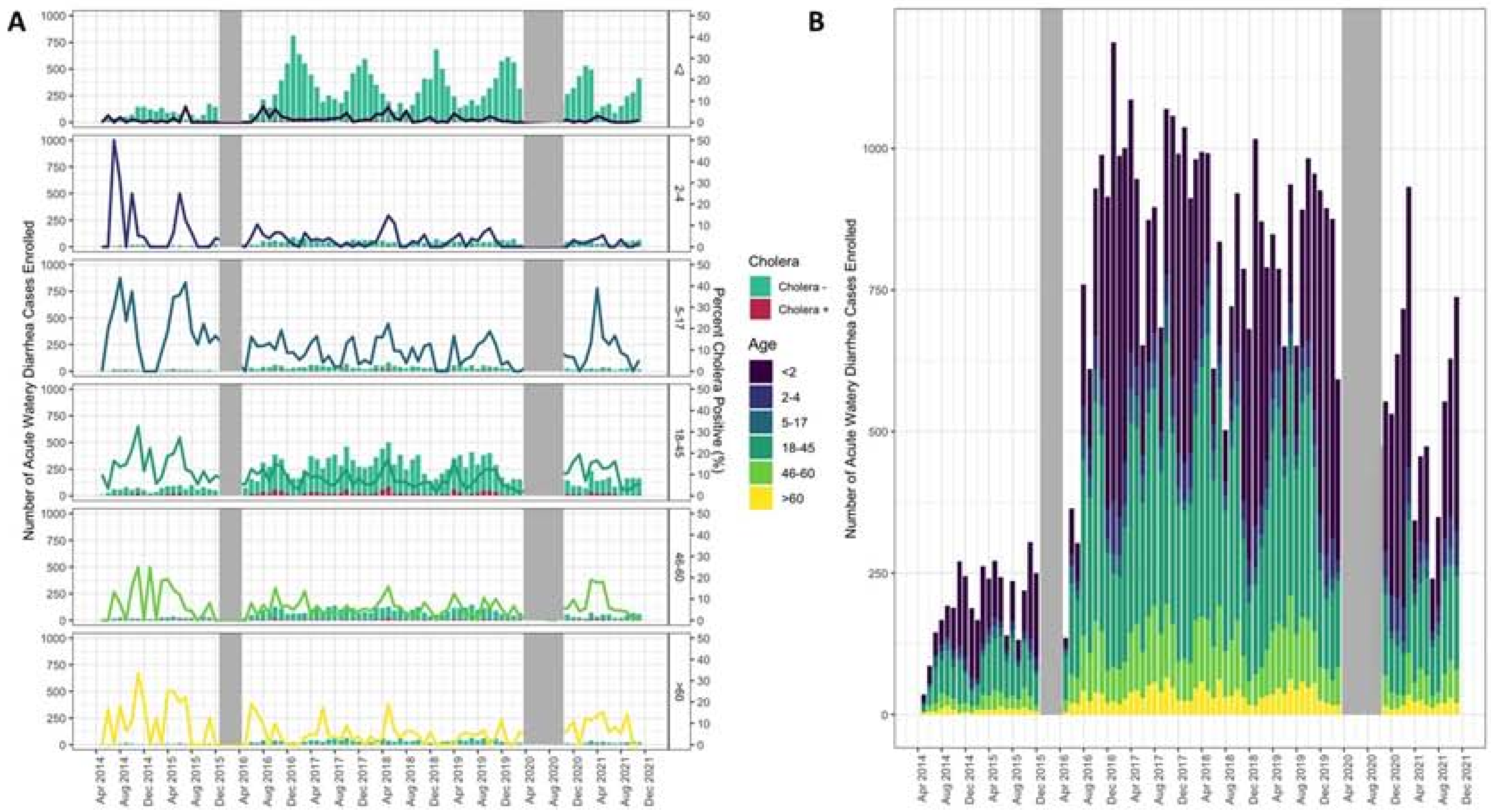

Supplemental Figure 4. A. Heat map of the average percent of suspected cases that are cholera positive by month and site (ordered from the northern end of Bangladesh to the southern), and the average number of suspected cholera cases enrolled by month from 2014-2021. Per the protocol, approximately 80 suspected cases (20 cases per week x 4 weeks per month) should have been enrolled from 2016-2021. Prior to 2016, approximately 40 suspected cases (10 cases per week x 4 weeks per month) were enrolled. B. Dot plot of the average percent of suspected cases that are cholera positive where each dot represents a unique sentinel site (ordered from the northern end of Bangladesh to the southern) categorized by age group (<5 vs. >5 years) and season: March-June (pre-monsoon), July-August (monsoon), Sep-Oct (post-monsoon), and Other (Nov-Feb).

A

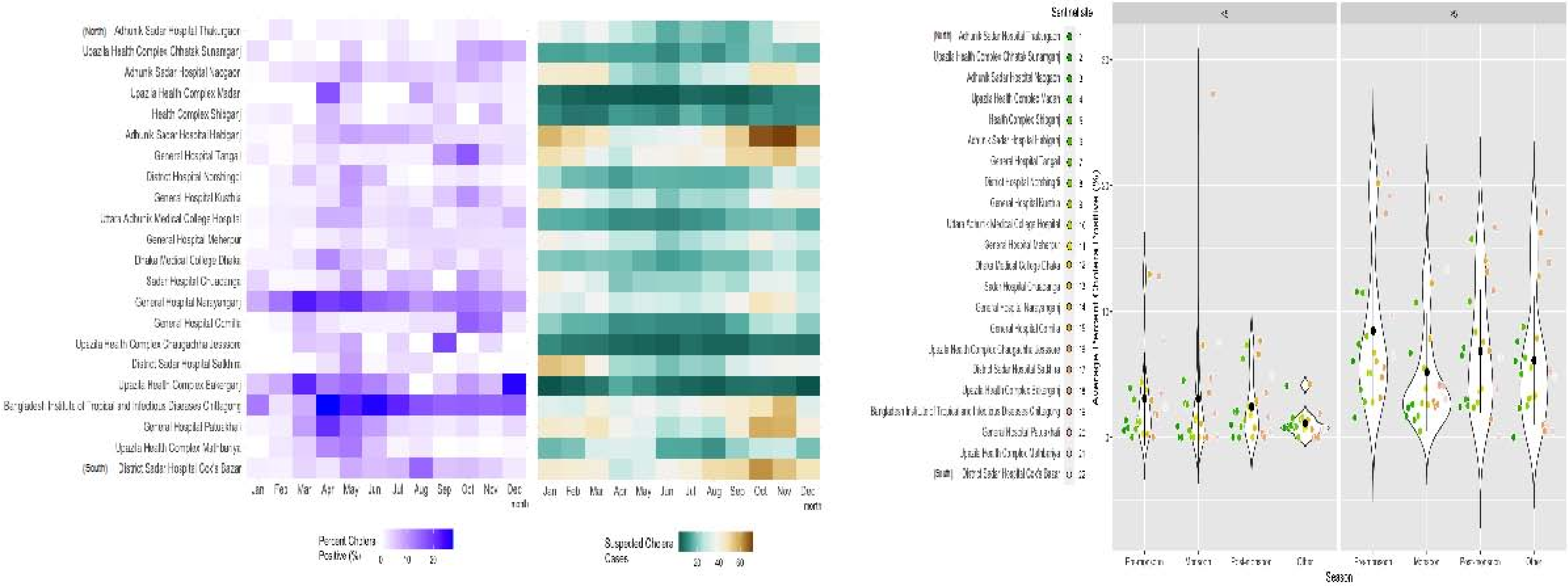

Supplemental Figure 5: Weekly distribution of suspected and confirmed cholera cases

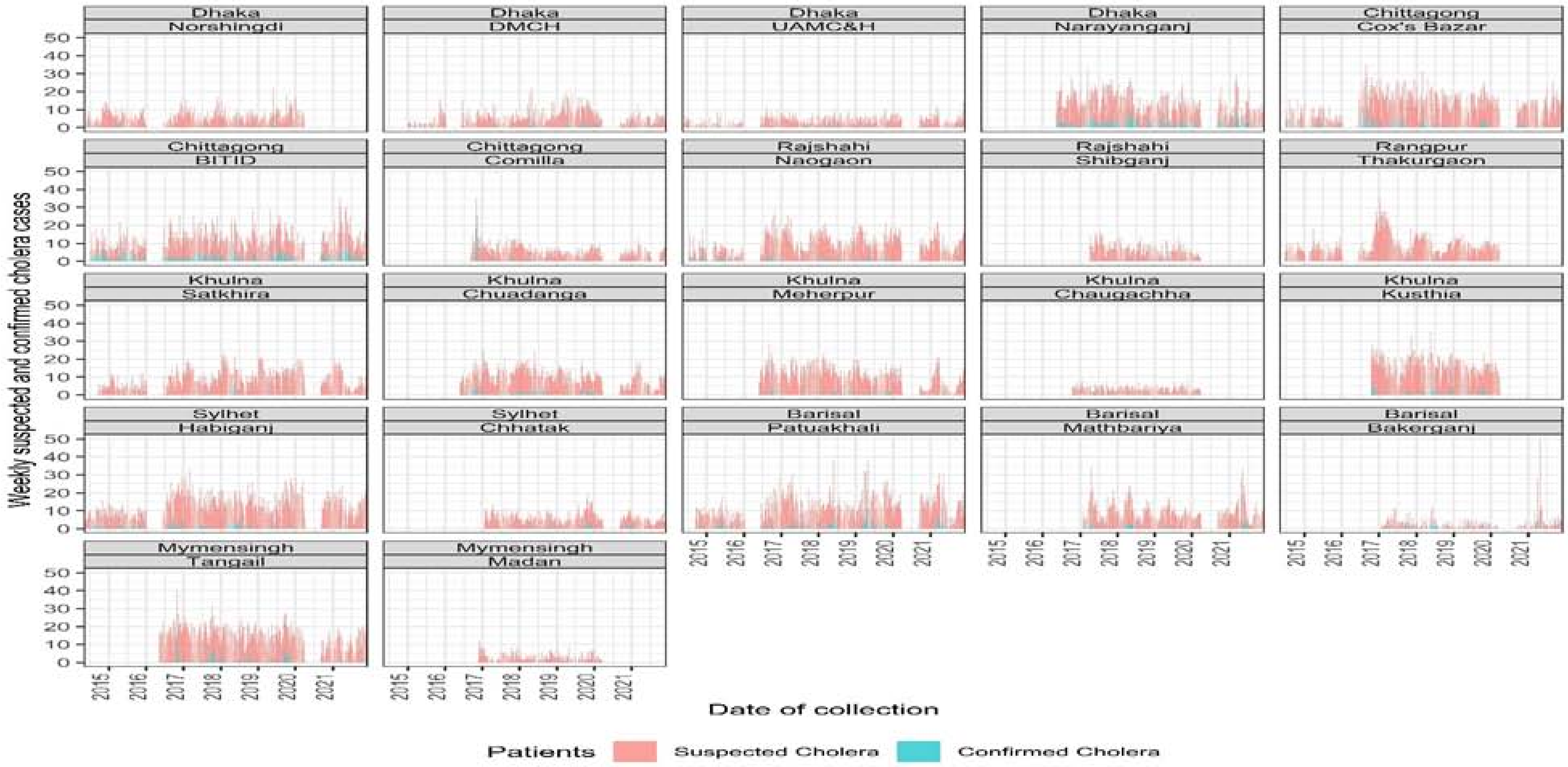

Supplemental Figure 6. Antibiotic susceptibility pattern by antibiotic and serotype for the period of 2014 to 2021

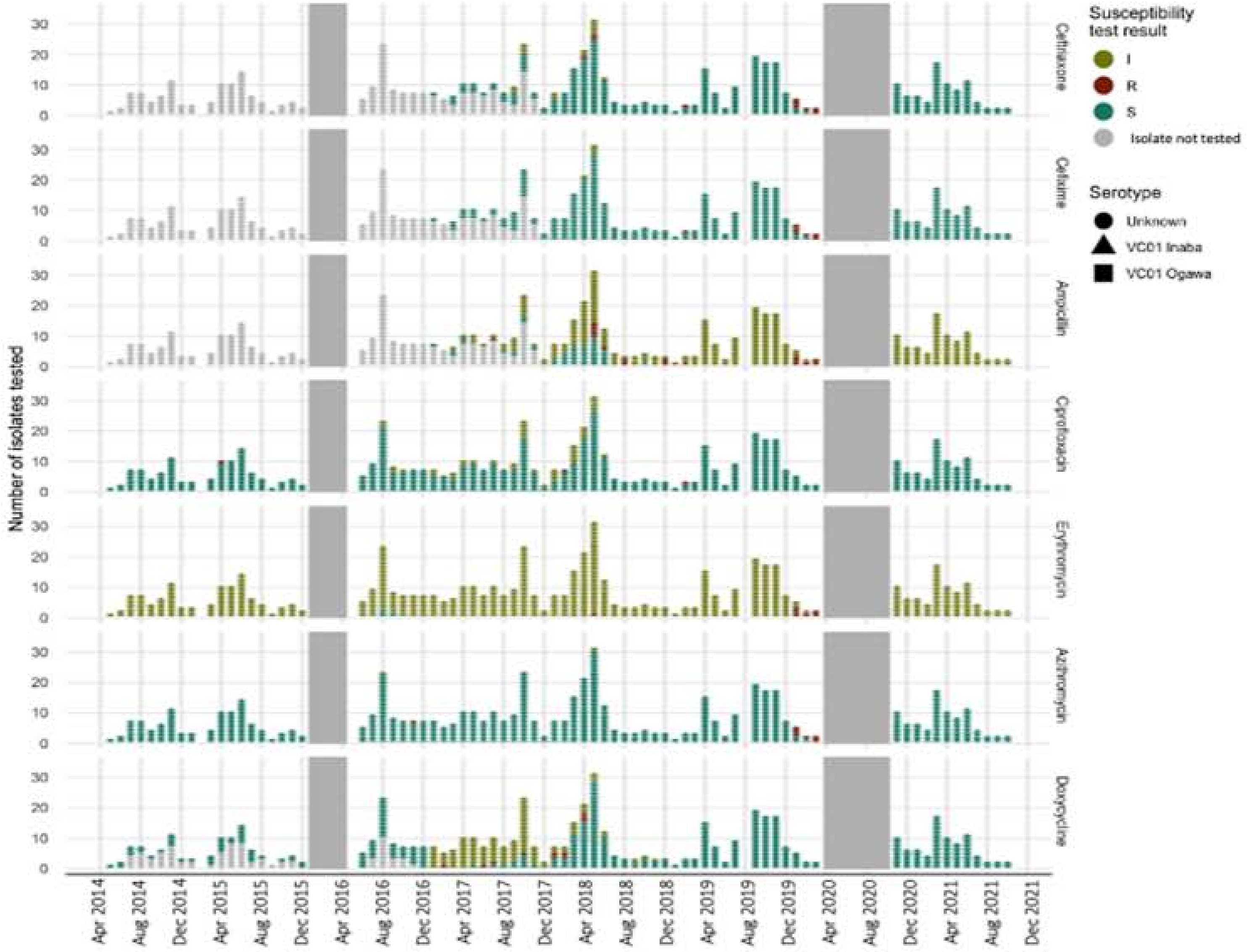

Ceftriaxone	Cefixime	Ampicillin	Ciprofloxacin	Erythromycin	Azithromycin	Doxycycline	Antibiotic resistance pattern	
95.9	97.3	36.5	90.5	0	100	73	S	2018
2.7	2.7	54.1	9.5	98.6	0	24.3	I	
1.4	0	9.5	0	1.4	0	2.7	R	
100	100	0	100	0	100	100	S	2019
0	0	100	0	100	0	0	I	
0	0	0	0	0	0	0	R	
100	80.7	0	100	0	80.7	100	S	2020
0	0	80.7	0	80.7	19.3	0	I	
0	19.3	19.3	0	19.3	0	0	R	
100	100	0	100	0	100	100	S	2021
0	0	100	0	100	0	0	I	
0	0	0	0	0	0	0	R	

Supplemental Figure 7. Antibiotic susceptibility pattern by antibiotic and serotype for the period of 2014 to 2021

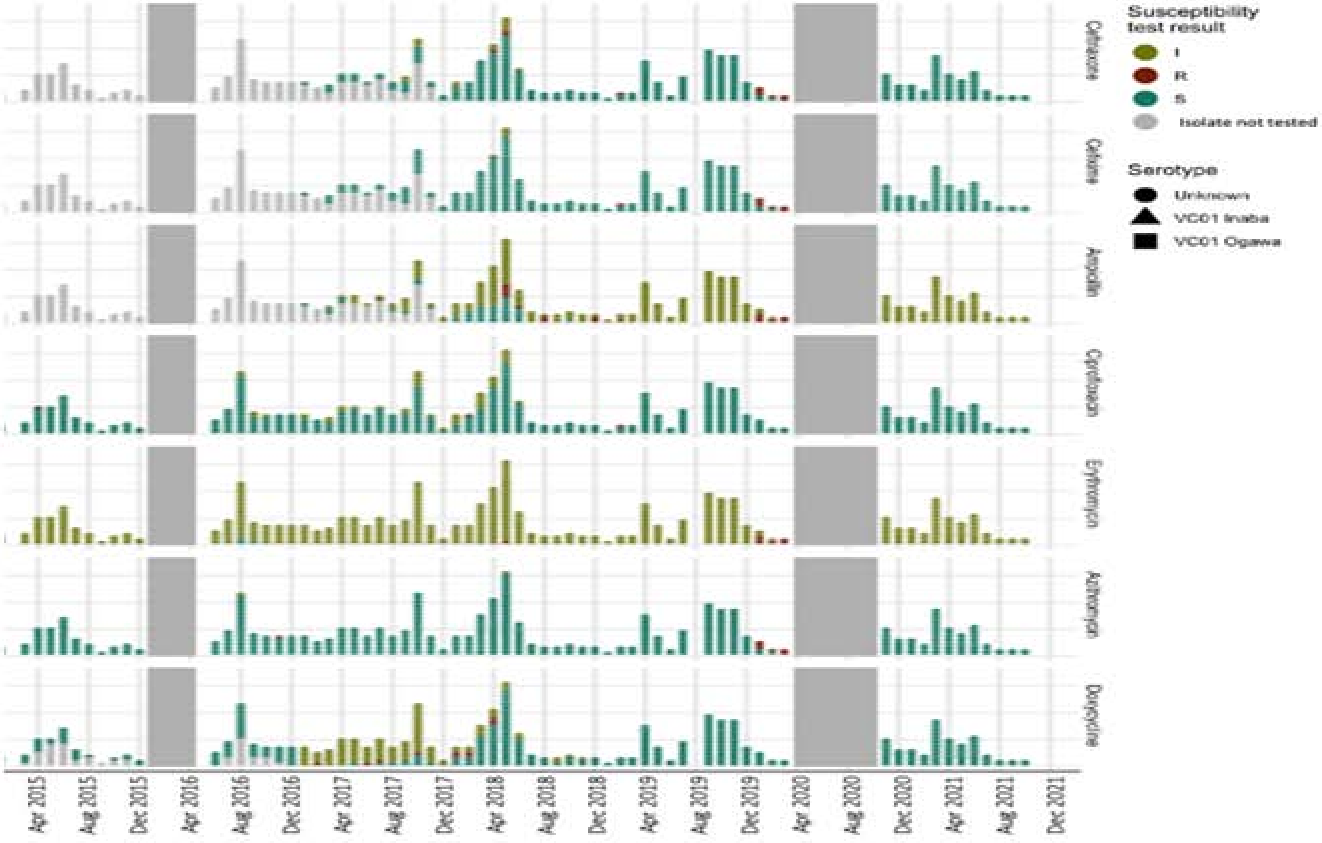

Ceftriaxone	Cefixime	Ampicillin	Ciprofloxacin	Erythromycin	Azithromycin	Doxycycline
95.9	97.3	36.5	90.5	0	100	73
2.7	2.7	54.1	9.5	98.6	0	24.3
1.4	0	9.5	0	1.4	0	2.7
100	100	0	100	0	100	100
0	0	100	0	100	0	0
0	0	0	0	0	0	0
100	80.7	0	100	0	80.7	100
0	0	80.7	0	80.7	19.3	0
0	19.3	19.3	0	19.3	0	0
100	100	0	100	0	100	100
0	0	100	0	100	0	0
0	0	0	0	0	0	0

Supplementary Table 1. Baseline characteristics of participants enrolled in the nationwide cholera surveillance by different Divisions in Bangladesh for the period of 2014 to 2021. Data from sentinel sites was grouped by Division.

Characteristic	Labels	Suspected case N = 51,414[1]	Dhaka, N = 7,825[1]	Chittagong, N = 9,006[1]	Rajshahi, N = 4,129[1]	Rangpur, N = 2,433[1]	Khulna, N = 11,663[1]	Sylhet, N = 5,748[1]	Barisal, N = 6,275[1]	Mymensingh, N = 4,335[1]
**Age (Years)**										
	<5, years	24,609 (48%)	2,390 (31%)	2,842 (32%)	2,201 (53%)	2,215 (91%)	5,795 (50%)	3,706 (64%)	3,242 (52%)	2,218 (51%)
	5-17, years	2,510 (4.9%)	411 (5.3%)	521 (5.8%)	163 (3.9%)	26 (1.1%)	587 (5.0%)	166 (2.9%)	311 (5.0%)	325 (7.5%)
	18-45, years	16,816 (33%)	3,473 (44%)	4,160 (46%)	1,155 (28%)	130 (5.3%)	3,512 (30%)	1,323 (23%)	1,754 (28%)	1,309 (30%)
	46-60, years	3,958 (7.7%)	788 (10%)	812 (9.0%)	333 (8.1%)	37 (1.5%)	955 (8.2%)	264 (4.6%)	451 (7.2%)	318 (7.3%)
	61+, years	3,521 (6.8%)	763 (9.8%)	671 (7.5%)	277 (6.7%)	25 (1.0%)	814 (7.0%)	289 (5.0%)	517 (8.2%)	165 (3.8%)
**Gender**										
	Female	23,840 (46%)	3,531 (45%)	4,563 (51%)	1,782 (43%)	944 (39%)	5,418 (46%)	2,398 (42%)	2,963 (47%)	2,241 (52%)
	Male	27,574 (54%)	4,294 (55%)	4,443 (49%)	2,347 (57%)	1,489 (61%)	6,245 (54%)	3,350 (58%)	3,312 (53%)	2,094 (48%)
**Occupation**										
	Service holder	3,534 (6.9%)	1,407 (18%)	1,182 (13%)	114 (2.8%)	14 (0.6%)	338 (2.9%)	84 (1.5%)	169 (2.7%)	226 (5.2%)

	House Wife	11,590 (23%)	1,920 (25%)	2,457 (27%)	855 (21%)	101 (4.2%)	2,655 (23%)	897 (16%)	1,542 (25%)	1,163 (27%)
	Agriculture worker	2,220 (4.3%)	186 (2.4%)	180 (2.0%)	293 (7.1%)	43 (1.8%)	1,014 (8.7%)	224 (3.9%)	210 (3.3%)	70 (1.6%)
	Business man	2,186 (4.3%)	564 (7.2%)	452 (5.0%)	211 (5.1%)	17 (0.7%)	500 (4.3%)	86 (1.5%)	250 (4.0%)	106 (2.4%)
	Labor/Worker/Driver	1,880 (3.7%)	363 (4.6%)	645 (7.2%)	154 (3.7%)	5 (0.2%)	261 (2.2%)	262 (4.6%)	123 (2.0%)	67 (1.5%)
	Student and Unemployed	3,836 (7.5%)	709 (9.1%)	949 (11%)	202 (4.9%)	22 (0.9%)	743 (6.4%)	385 (6.7%)	574 (9.1%)	252 (5.8%)
	Child(up to 10 years)	25,785 (50%)	2,536 (32%)	3,064 (34%)	2,282 (55%)	2,228 (92%)	6,101 (52%)	3,779 (66%)	3,380 (54%)	2,415 (56%)
	Others	383 (0.7%)	140 (1.8%)	77 (0.9%)	18 (0.4%)	3 (0.1%)	51 (0.4%)	31 (0.5%)	27 (0.4%)	36 (0.8%)
**Education Status**										
	Illiterate	7,034 (14%)	837 (11%)	1,393 (15%)	598 (14%)	11 (0.5%)	2,083 (18%)	925 (16%)	560 (8.9%)	627 (14%)
	Up to class VIII	12,565 (24%)	2,861 (37%)	3,269 (36%)	827 (20%)	94 (3.9%)	2,541 (22%)	883 (15%)	1,353 (22%)	737 (17%)
	Secondary and above	6,268 (12%)	1,466 (19%)	1,358 (15%)	413 (10%)	109 (4.5%)	1,139 (9.8%)	198 (3.4%)	1,066 (17%)	519 (12%)

	Others (Child,etc.)	25,547 (50%)	2,661 (34%)	2,986 (33%)	2,291 (55%)	2,219 (91%)	5,900 (51%)	3,742 (65%)	3,296 (53%)	2,452 (57%)
**Duration of Diarrhea**	Median (Q1, Q3)	2 (1,3)	2 (1,3)	2 (1,2)	2 (1,3)	4 (3,4)	2 (1,3)	3 (1,4)	2 (1,3)	2 (1,3)
**Number of purging in last 24hours**	Median (Q1, Q3)	15 (10,20)	16 (10,22)	12 (9,15)	18 (14,25)	14 (10,15)	14 (8,20)	20 (15,25)	13 (10,16)	20 (15,30)
**Nature of Stool**										
	Loose watery	36,073 (70%)	5,960 (76%)	6,050 (67%)	1,265 (31%)	1,373 (56%)	8,216 (70%)	5,070 (88%)	4,267 (68%)	3,872 (89%)
	Rice watery	14,267 (28%)	1,187 (15%)	2,719 (30%)	2,823 (68%)	1,058 (43%)	3,447 (30%)	678 (12%)	1,892 (30%)	463 (11%)
	Formed	1,074 (2.1%)	678 (8.7%)	237 (2.6%)	41 (1.0%)	2 (<0.1%)	0 (0%)	0 (0%)	116 (1.8%)	0 (0%)
**Vomiting**	Yes	33,048 (64%)	6,687 (85%)	6,770 (75%)	2,547 (62%)	1,865 (77%)	5,384 (46%)	2,136 (37%)	4,688 (75%)	2,971 (69%)
**Dehydration**										
	No	9,457 (18%)	559 (7.1%)	1,250 (14%)	179 (4.3%)	238 (9.8%)	1,167 (10%)	2,578 (45%)	2,347 (37%)	1,139 (26%)
	Some	34,151 (66%)	4,692 (60%)	6,545 (73%)	3,375 (82%)	902 (37%)	9,922 (85%)	1,966 (34%)	3,789 (60%)	2,960 (68%)
	Severe	7,806 (15%)	2,574 (33%)	1,211 (13%)	575 (14%)	1,293 (53%)	574 (4.9%)	1,204 (21%)	139 (2.2%)	236 (5.4%)
**Abdominal Cramp**	Yes	29,924 (58%)	6,221 (80%)	6,125 (68%)	2,425 (59%)	179 (7.4%)	5,883 (50%)	1,686 (29%)	4,061 (65%)	3,344 (77%)

**Fever**	Yes	29,497 (57%)	6,258 (80%)	5,119 (57%)	1,944 (47%)	1,493 (61%)	6,377 (55%)	3,368 (59%)	2,107 (34%)	2,831 (65%)
**Tap water**	Yes	12,634 (25%)	4,355 (56%)	3,802 (42%)	1,389 (34%)	4 (0.2%)	680 (5.8%)	185 (3.2%)	966 (15%)	1,253 (29%)
**Tube well**	Yes	42,457 (83%)	4,737 (61%)	5,495 (61%)	3,908 (95%)	2,427 (100%)	11,212 (96%)	5,677 (99%)	4,855 (77%)	4,146 (96%)
**Bottle water**	Yes	4,434 (8.6%)	579 (7.4%)	658 (7.3%)	125 (3.0%)	53 (2.2%)	234 (2.0%)	43 (0.7%)	2,372 (38%)	370 (8.5%)
**Water treated by Boiled/Filtered/Chemical**	Yes	7,818 (15%)	1,667 (21%)	1,360 (15%)	448 (11%)	51 (2.1%)	252 (2.2%)	328 (5.7%)	2,328 (37%)	1,384 (32%)
**Take food from road side**	Yes	14,166 (28%)	1,084 (14%)	2,838 (32%)	2,622 (64%)	19 (0.8%)	3,161 (27%)	2,039 (35%)	1,868 (30%)	535 (12%)
**Take food from large gatherings**	Yes	5,413 (11%)	370 (4.7%)	1,756 (19%)	1,209 (29%)	9 (0.4%)	856 (7.3%)	733 (13%)	388 (6.2%)	92 (2.1%)
**Any one of neighbor have the same disease**	Yes	7,741 (15%)	124 (1.6%)	1,858 (21%)	561 (14%)	22 (0.9%)	561 (4.8%)	1,000 (17%)	3,130 (50%)	485 (11%)
**Use of antibiotic for current illness**	Yes	22,875 (44%)	2,162 (28%)	4,381 (49%)	1,421 (34%)	1,132 (47%)	5,133 (44%)	3,952 (69%)	1,594 (25%)	3,100 (72%)
**Cholera**										
	Negative	48,743 (95%)	7,250 (93%)	8,134 (90%)	3,984 (96%)	2,412 (99%)	11,340 (97%)	5,539 (96%)	5,924 (94%)	4,160 (96%)
	Positive	2,671 (5.2%)	575 (7.3%)	872 (9.7%)	145 (3.5%)	21 (0.9%)	323 (2.8%)	209 (3.6%)	351 (5.6%)	175 (4.0%)
**Season**										

	Pre-monsoon (March-June)	14,918 (29%)	2,301 (29%)	2,534 (28%)	1,130 (27%)	788 (32%)	3,282 (28%)	1,507 (26%)	2,223 (35%)	1,153 (27%)
	Monsoon (July-August)	7,164 (14%)	1,114 (14%)	1,444 (16%)	651 (16%)	296 (12%)	1,507 (13%)	809 (14%)	673 (11%)	670 (15%)
	Post-monsoon (Sept-Oct)	9,627 (19%)	1,489 (19%)	1,818 (20%)	754 (18%)	285 (12%)	2,002 (17%)	1,198 (21%)	1,239 (20%)	842 (19%)
	Others (Nov-Feb)	19,705 (38%)	2,921 (37%)	3,210 (36%)	1,594 (39%)	1,064 (44%)	4,872 (42%)	2,234 (39%)	2,140 (34%)	1,670 (39%)

[1]n (%)

Supplementary Table 2. Baseline characteristics of cholera positive participants enrolled in the nationwide cholera surveillance by Division, Bangladesh for the period of 2014 to 2021. Data from sentinel sites was grouped by Division.

Characteristic	Labels	Culture confirmed , N = 2,671[1]	Dhaka, N = 575[1]	Chittagong, N = 872[1]	Rajshahi, N = 145[1]	Rangpur, N = 21[1]	Khulna, N = 323[1]	Sylhet, N = 209[1]	Barisal, N = 351[1]	Mymensingh, N = 175[1]
**Season**										
	Pre-monsoon	1,060 (40%)	265 (46%)	286 (33%)	44 (30%)	6 (29%)	99 (31%)	70 (33%)	264 (75%)	26 (15%)
	Monsoon	409 (15%)	65 (11%)	192 (22%)	23 (16%)	3 (14%)	54 (17%)	42 (20%)	19 (5.4%)	11 (6.3%)
	Post-monsoon	595 (22%)	103 (18%)	187 (21%)	38 (26%)	4 (19%)	89 (28%)	44 (21%)	28 (8.0%)	102 (58%)
	Others	607 (23%)	142 (25%)	207 (24%)	40 (28%)	8 (38%)	81 (25%)	53 (25%)	40 (11%)	36 (21%)
**Age (Years)**										
	<5, years	379 (14%)	50 (8.7%)	87 (10.0%)	19 (13%)	16 (76%)	59 (18%)	58 (28%)	57 (16%)	33 (19%)
	5-17, years	282 (11%)	46 (8.0%)	112 (13%)	13 (9.0%)	0 (0%)	24 (7.4%)	30 (14%)	36 (10%)	21 (12%)
	18-45, years	1,554 (58%)	367 (64%)	528 (61%)	87 (60%)	4 (19%)	186 (58%)	98 (47%)	184 (52%)	100 (57%)
	46-60, years	243 (9.1%)	68 (12%)	76 (8.7%)	12 (8.3%)	0 (0%)	33 (10%)	7 (3.3%)	37 (11%)	10 (5.7%)

	61+, years	213 (8.0%)	44 (7.7%)	69 (7.9%)	14 (9.7%)	1 (4.8%)	21 (6.5%)	16 (7.7%)	37 (11%)	11 (6.3%)
**Gender**										
	Female	1,261 (47%)	226 (39%)	438 (50%)	58 (40%)	7 (33%)	157 (49%)	96 (46%)	178 (51%)	101 (58%)
	Male	1,410 (53%)	349 (61%)	434 (50%)	87 (60%)	14 (67%)	166 (51%)	113 (54%)	173 (49%)	74 (42%)
**Occupation**										
	Service holder	455 (17%)	199 (35%)	178 (20%)	12 (8.3%)	0 (0%)	14 (4.3%)	5 (2.4%)	23 (6.6%)	24 (14%)
	House Wife	782 (29%)	124 (22%)	245 (28%)	40 (28%)	1 (4.8%)	115 (36%)	56 (27%)	135 (38%)	66 (38%)
	Agriculture worker	139 (5.2%)	17 (3.0%)	18 (2.1%)	16 (11%)	1 (4.8%)	44 (14%)	18 (8.6%)	21 (6.0%)	4 (2.3%)
	Businessman	190 (7.1%)	46 (8.0%)	58 (6.7%)	16 (11%)	1 (4.8%)	24 (7.4%)	6 (2.9%)	27 (7.7%)	12 (6.9%)
	Labor/Worker/Driver	244 (9.1%)	59 (10%)	116 (13%)	17 (12%)	1 (4.8%)	9 (2.8%)	19 (9.1%)	14 (4.0%)	9 (5.1%)
	Student and Unemployed	343 (13%)	55 (9.6%)	109 (12%)	19 (13%)	1 (4.8%)	46 (14%)	32 (15%)	62 (18%)	19 (11%)
	Child(up to 10 years)	493 (18%)	66 (11%)	140 (16%)	25 (17%)	16 (76%)	69 (21%)	70 (33%)	67 (19%)	40 (23%)
	Others	25 (0.9%)	9 (1.6%)	8 (0.9%)	0 (0%)	0 (0%)	2 (0.6%)	3 (1.4%)	2 (0.6%)	1 (0.6%)
**Education Status**										

	Illiterate	511 (19%)	88 (15%)	166 (19%)	15 (10%)	0 (0%)	88 (27%)	65 (31%)	52 (15%)	37 (21%)
	Up to class VIII	1,197 (45%)	311 (54%)	468 (54%)	66 (46%)	2 (9.5%)	109 (34%)	73 (35%)	116 (33%)	52 (30%)
	Secondary and above	518 (19%)	107 (19%)	132 (15%)	40 (28%)	3 (14%)	62 (19%)	8 (3.8%)	123 (35%)	43 (25%)
	Others (Child,etc.)	445 (17%)	69 (12%)	106 (12%)	24 (17%)	16 (76%)	64 (20%)	63 (30%)	60 (17%)	43 (25%)
**Duration of Diarrhea**	Median (Q1, Q3)	2 (1,2)	1 (0.6,2)	1 (1,2)	2 (1,2)	4 (3,4)	2 (1,2.5)	2 (1,2)	1 (1,2)	2 (2,3)
**Number of purging in last 24hours**	Median (Q1, Q3)	16 (12,22)	21 (16,25)	13 (10,18)	20 (16,25)	15 (13,15)	15 (10,20)	20 (15,25)	15 (12,18)	20 (15,30)
**Nature of Stool**										
	Loose watery	1,746 (65%)	311 (54%)	609 (70%)	55 (38%)	7 (33%)	228 (71%)	153 (73%)	238 (68%)	145 (83%)
	Rice watery	901 (34%)	256 (45%)	253 (29%)	89 (61%)	14 (67%)	95 (29%)	56 (27%)	108 (31%)	30 (17%)
	Formed	24 (0.9%)	8 (1.4%)	10 (1.1%)	1 (0.7%)	0 (0%)	0 (0%)	0 (0%)	5 (1.4%)	0 (0%)
**Vomiting**	Yes	2,012 (75%)	535 (93%)	636 (73%)	106 (73%)	16 (76%)	206 (64%)	117 (56%)	260 (74%)	136 (78%)
**Dehydration**										
	No	432 (16%)	16 (2.8%)	199 (23%)	24 (17%)	1 (4.8%)	21 (6.5%)	55 (26%)	85 (24%)	31 (18%)
	Some	1,465	170	475 (54%)	97 (67%)	5 (24%)	262	98	239	119 (68%)

		(55%)	(30%)				(81%)	(47%)	(68%)	
	Severe	774 (29%)	389 (68%)	198 (23%)	24 (17%)	15 (71%)	40 (12%)	56 (27%)	27 (7.7%)	25 (14%)
**Abdominal Cramp**	Yes	1,861 (70%)	523 (91%)	525 (60%)	92 (63%)	4 (19%)	194 (60%)	104 (50%)	282 (80%)	137 (78%)
**Fever**	Yes	1,260 (47%)	496 (86%)	295 (34%)	41 (28%)	11 (52%)	167 (52%)	108 (52%)	74 (21%)	68 (39%)
**Tap water**	Yes	1,027 (38%)	360 (63%)	430 (49%)	57 (39%)	0 (0%)	33 (10%)	7 (3.3%)	66 (19%)	74 (42%)
**Tube well**	Yes	1,932 (72%)	360 (63%)	425 (49%)	137 (94%)	21 (100%)	310 (96%)	207 (99%)	307 (87%)	165 (94%)
**Bottle water**	Yes	265 (9.9%)	23 (4.0%)	77 (8.8%)	12 (8.3%)	2 (9.5%)	11 (3.4%)	1 (0.5%)	129 (37%)	10 (5.7%)
**Water treated by Boiled/Filtered/Chemical**	Yes	518 (19%)	99 (17%)	182 (21%)	12 (8.3%)	0 (0%)	13 (4.0%)	12 (5.7%)	128 (36%)	72 (41%)
**Take food from road side**	Yes	843 (32%)	70 (12%)	367 (42%)	83 (57%)	0 (0%)	112 (35%)	71 (34%)	106 (30%)	34 (19%)
**Take food from large gatherings**	Yes	370 (14%)	20 (3.5%)	199 (23%)	39 (27%)	0 (0%)	36 (11%)	31 (15%)	37 (11%)	8 (4.6%)
**Any one of neighbor have the same disease**	Yes	482 (18%)	7 (1.2%)	181 (21%)	16 (11%)	1 (4.8%)	21 (6.5%)	37 (18%)	195 (56%)	24 (14%)
**Use of antibiotic for current illness**	Yes	1,053 (39%)	129 (22%)	407 (47%)	39 (27%)	10 (48%)	134 (41%)	124 (59%)	84 (24%)	126 (72%)

[1]n (%)

Supplementary Table 3: Mean percentage of suspected cases that are culture confirmed positive for *Vibrio cholerae* by sentinel sites and averaged across Divisions from 2014 to 2021

**Sentinel Site**	**Division**	**Proportion of AWD that are cholera + (%)**	**Division average cholera positivity (%)**
General Hospital Patuakhali	Barisal	5.5	5.6
Upazila Health Complex Mathbariya		4.1	
Upazila Health Complex Bakerganj		11.2	
District Sadar Hospital Cox's Bazar	Chittagong	5.3	9.7
Bangladesh Institute of Tropical and Infectious Diseases Chittagong		16.7	
General Hospital Comilla		5.4	
District Hospital Norshingdi	Dhaka	2.3	7.4
Dhaka Medical College Dhaka		3.4	
Uttara Adhunik Medical College Hospital		3.5	
General Hospital Tangail		4.2	
General Hospital Narayanganj		14.0	
District Sadar Hospital Satkhira	Khulna	1.3	2.8
Sadar Hospital Chuadanga		4.1	
General Hospital Meherpur		2.3	
Upazila Health Complex ChaugachhaJesssore		3.1	
General Hospital Kusthia		3.6	
Upazila Health Complex Madan	Mymensingh	2.7	4.0
AdhunikSadar Hospital Naogaon	Rajshahi	3.9	3.5
Health Complex Shibganj		2.3	
AdhunikSadar Hospital Thakurgaon	Rangpur	0.9	0.9
AdhunikSadar Hospital Habiganj	Sylhet	3.7	3.6
Upazila Health Complex ChhatakSunamganj		3.6	

Supplement Table 4. Associations of confirmed cholera with sociodemographic, clinical features and possible exposures using Generalized Estimating Equation (GEE) across the sentinel sites for the entire the study period and stratified by age using the following groups: <5, ≥5-17 and ≥ 18 years of age. Age groups were chosen to match the enrollment scheme. The95% CIs were calculated using robust standard errors from the GEE models. P-values are not corrected using the Bonferroni correction.

**Characteristics**	**Labels**	**Age: <5, years old**		**Age: 5-17, years old**		**Age: 18+, years old**	
		OR (95% CI)	p-value	OR (95% CI)	p-value	OR (95% CI)	p-value
**Season**	Others (ref)	1.00		1.00		1.00	
	Pre-monsoon	2.42 (1.50, 3.93)	0.000	1.76 (1.26,2.26)	0.001	1.43 (1.13, 1.80)	0.003
	Monsoon	2.94 (1.57, 5.51)	0.001	1.58 (0.96, 2.58)	0.070	0.95 (0.73, 1.24)	0.698
	Post-monsoon	2.29 (1.37, 3.85)	0.002	1.53 (1.15, 2.03)	0.004	1.17 (0.92, 1.48)	0.194
**Gender**	Male	0.92 (0.77, 1.10)	0.386	1.02 (0.83, 1.24)	0.872	1.3 (1.17, 1.45)	0.000
**Occupation**	House wife (ref)					1.00	
	Service holder					1.86 (1.52, 2.28)	0.000
	Agriculture worker					0.94 (0.72, 1.22)	0.625
	Businessman					1.3 (1.11, 1.51)	0.001
	Labor/Worker/Driver					1.89 (1.51, 2.38)	0.000
	Student and Unemployed					1.16 (0.98, 1.37)	0.076
	Others_ocu					1 (0.60, 1.64)	0.989
**Education**	Illiterate (ref)			1.00		1.00	
	Up to class VIII			0.62 (0.42, 0.91)	0.015	1.25 (1.02, 1.52)	0.029

	Secondary and above			0.43(0.24, 0.79)	0.007	1.14 (0.89, 1.47)	0.296
	Others (Child,etc.)			0.25 (0.17, 0.38)	0.000	0.93 (0.56, 1.55)	0.793
**Duration of Diarrhea**	Number of days	0.82 (0.68, 0.98)	0.030	0.93 (0.85, 1.01)	0.095	0.83 (0.77, 0.89)	0.000
**Number of purging**	Number of times	1.01 (1.00, 1.03))	0.022	1.02 (1.00, 1.03)	0.030	1.02 (1.01, 1.03)	0.003
**Nature of Stool**	Loose watery (ref)	1.00		1.00		1.00	
	Rice watery stool	1.19 (0.68, 2.09)	0.549	1.2 (0.62, 2.32)	0.585	1.56 (0.93, 2.62)	0.091
	Formed stool	1.19 (0.61, 2.33)	0.607	0.57 (0.32, 1.01)	0.055	0.31 (0.18, 0.56)	0.000
**Vomiting**		1.42 (1.12, 1.80)	0.003	1.38 (1.02, 1.89)	0.039	1.58 (1.10, 2.27)	0.013
**Dehydration**	None	1.00		1.00		1.00	
	Some	0.93 (0.73, 1.17)	0.529	0.7 (0.31, 1.56)	0.380	0.67 (0.51, 0.89)	0.006
	Severe	1.5(0.86, 2.62))	0.154	1.72 (0.77, 3.83)	0.187	1.4 (0.91, 2.16)	0.125
**Abdominal Cramp**		1.64 (1.33, 2.03)	0.000	0.68 (0.46, 1.00)	0.049	0.82 (0.57, 1.19)	0.295
**Fever**		1.03 (0.76, 1.39)	0.855	0.47 (0.30, 0.74)	0.001	0.71 (0.43, 1.18)	0.188
**Tap water**		1.72 (1.26, 2.35)	0.001	0.99 (0.66, 1.47)	0.945	1.52 (1.20, 1.93)	0.001
**Tube well**		0.59 (0.41, 085)	0.005	0.78 (0.44, 1.38)	0.388	0.67 (0.47, 0.96)	0.031
**Bottle water**		1.06 (0.75, 1.51)	0.723	1.08 (0.58, 1.99)	0.814	1.18 (0.85, 1.65)	0.324
**Water treated by Boiled/Filtered/Chemical**		1.46 (1.09, 1.95)	0.010	0.89 (0.63, 1.25)	0.493	1.41 (1.10, 1.81)	0.006
**Take food from road side**		1.22 (0.82, 1.82)	0.325	0.97 (0.72, 1.30)	0.821	1.06 (0.79, 1.41)	0.710
**Take food from large gatherings**		0.74 (0.40, 1.38)	0.343	1.41 (1.03, 1.93)	0.031	1.03 (0.81, 1.30)	0.817
**Any one of neighbor have the same disease**		1.13 (0.78, 1.62)	0.516	1.83 (1.21, 2.76)	0.004	1.5 (1.06, 2.12)	0.023
